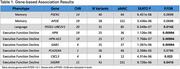# Rare variant associations with late‐life cognitive performance

**DOI:** 10.1002/alz70855_105742

**Published:** 2025-12-24

**Authors:** Alexandra N. Regelson, Derek B. Archer, Alaina Durant, Shubhabrata Mukherjee, Michael L. Lee, Seo‐Eun Choi, Phoebe Scollard, Emily H. Trittschuh, Jesse Mez, William S Bush, Amanda Kuzma, Michael L Cuccaro, Carlos Cruchaga, Lindsay A. Farrer, Li‐San Wang, Gerald D. Schellenberg, Richard Mayeux, Walter W. Kukull, C. Dirk Keene, Andrew J. Saykin, Sterling C Johnson, Corinne D. Engelman, David A. A. Bennett, Lisa L. Barnes, Eric B Larson, Kwangsik Nho, Alison M. Goate, Alan E. Renton, Edoardo Marcora, Brian Fulton‐Howard, Tulsi Patel, Shannon L Risacher, Anita L. DeStefano, Julie A Schneider, Mohamad Habes, Sudha Seshadri, Claudia L Satizabal, Pauline Maillard, Arthur W. Toga, Karen Crawford, Duygu Tosun, Jeffery M Vance, Elizabeth C. Mormino, Charles Decarli, Thomas J. Montine, Gary W Beecham, Sarah Biber, Philip L. De Jager, Vilas Menon, Annie J. Lee, Adam Brickman, Badri N. Vardarajan, Christiane Reitz, Jennifer J. Manly, Jason M Fletcher, Miguel Arce Renteria, Jason Song, Qiongshi Lu, Paul K Crane, Timothy J. Hohman, Logan Dumitrescu, The Alzheimer's Disease Neuroimaging Initiative (ADNI) and The Alzheimer's Disease Sequencing Project (ADSP)

**Affiliations:** ^1^ Vanderbilt Genetics Institute, Vanderbilt University Medical Center, Nashville, TN, USA; ^2^ Vanderbilt Memory & Alzheimer's Center, Vanderbilt University Medical Center, Nashville, TN, USA; ^3^ Vanderbilt Genetics Institute, Vanderbilt University Medical Center, Nashville, TN, USA; ^4^ Vanderbilt University Medical Center, Nashville, TN, USA; ^5^ Department of Medicine, University of Washington, Seattle, WA, USA; ^6^ University of Washington, School of Medicine, Seattle, WA, USA; ^7^ Department of Psychiatry and Behavioral Sciences, University of Washington School of Medicine, Seattle, WA, USA; ^8^ VA Puget Sound Health Care System, Seattle, WA, USA; ^9^ Boston University Chobanian & Avedisian School of Medicine, Boston, MA, USA; ^10^ Cleveland Institute for Computational Biology, Department of Population and Quantitative Health Sciences, Case Western Reserve University, Cleveland, OH, USA; ^11^ Penn Neurodegeneration Genomics Center, Department of Pathology and Laboratory Medicine, Perelman School of Medicine, University of Pennsylvania, Philadelphia, PA, USA; ^12^ John P. Hussman Institute for Human Genomics, University of Miami Miller School of Medicine, Miami, FL, USA; ^13^ NeuroGenomics and Informatics Center, Washington University School of Medicine, St. Louis, MO, USA; ^14^ Washington University School of Medicine, St. Louis, MO, USA; ^15^ Biomedical Genetics, Department of Medicine, Boston University Medical School, Boston, MA, USA; ^16^ Department of Neurology, Boston University Chobanian & Avedisian School of Medicine, Boston, MA, USA; ^17^ Department of Biostatistics, Boston University School of Public Health, Boston, MA, USA; ^18^ Penn Neurodegeneration Genomics Center, Department of Pathology and Laboratory Medicine, University of Pennsylvania Perelman School of Medicine, Philadelphia, PA, USA; ^19^ Columbia University, New York, NY, USA; ^20^ Taub Institute for Research on Alzheimer's Disease and the Aging Brain, Columbia University, New York, NY, USA; ^21^ The Institute for Genomic Medicine, Columbia University Medical Center, New York, NY, USA; ^22^ Department of Epidemiology, School of Public Health, University of Washington, Seattle, WA, USA; ^23^ Department of Laboratory Medicine and Pathology, University of Washington, Seattle, WA, USA; ^24^ Department of Medical and Molecular Genetics, School of Medicine, Indiana University, Indianapolis, IN, USA; ^25^ Department of Radiology and Imaging Sciences, Center for Neuroimaging, School of Medicine, Indiana University School of Medicine, Indianapolis, IN, USA; ^26^ Wisconsin Alzheimer's Disease Research Center, School of Medicine and Public Health, University of Wisconsin‐Madison, Madison, WI, USA; ^27^ Wisconsin Alzheimer's Disease Research Center, University of Wisconsin School of Medicine and Public Health, Madison, WI, USA; ^28^ Rush Alzheimer's Disease Center, Rush University Medical Center, Chicago, IL, USA; ^29^ Indiana Alzheimer's Disease Research Center, Indiana University School of Medicine, Indianapolis, IN, USA; ^30^ Ronald M. Loeb Center for Alzheimer's Disease, Icahn School of Medicine at Mount Sinai, New York, NY, USA; ^31^ Department of Radiology and Imaging Sciences, Indiana University School of Medicine, Indianapolis, IN, USA; ^32^ Glenn Biggs Institute for Alzheimer's & Neurodegenerative Diseases, University of Texas Health Sciences Center at San Antonio, San Antonio, TX, USA; ^33^ Glenn Biggs Institute for Alzheimer's & Neurodegenerative Diseases, University of Texas Health Science Center, San Antonio, TX, USA; ^34^ Department of Neurology and Center for Neuroscience, University of California, Davis, Davis, CA, USA; ^35^ University of Southern California Stevens Neuroimaging and Informatics Institute, Keck School of Medicine, University of Southern California, Los Angeles, CA, USA; ^36^ Department of Radiology and Biomedical Imaging, University of California, San Francisco, San Francisco, CA, USA; ^37^ University of Miami Miller School of Medicine, Miami, FL, USA; ^38^ Department of Neurology and Neurological Sciences, Stanford University School of Medicine, Stanford, CA, USA; ^39^ Department of Neurology & Imaging of Dementia and Aging Laboratory, University of California Davis, Sacramento, CA, USA; ^40^ Department of Pathology, Stanford University School of Medicine, Stanford, CA, USA; ^41^ John T. Macdonald Foundation Department of Human Genetics, University of Miami Miller School of Medicine, Miami, FL, USA; ^42^ National Alzheimer's Coordinating Center, University of Washington, Seattle, WA, USA; ^43^ Center for Translational & Computational Neuroimmunology, Columbia University Irving Medical Center, New York, NY, USA; ^44^ Department of Neurology, Columbia University Medical Center, New York, NY, USA; ^45^ G. H. Sergievsky Center, Vagelos College of Physicians and Surgeons, Columbia University, New York, NY, USA; ^46^ Department of Neurology, Vagelos College of Physicians and Surgeons, Columbia University, New York, NY, USA; ^47^ Department of Neurology, Columbia University, New York, NY, USA; ^48^ Department of Neurology, Gertrude H. Sergievsky Center, Taub Institute for Research on Alzheimer's Disease and The Aging Brain, Columbia University Medical Center, New York, NY, USA; ^49^ G.H. Sergievsky Center, Vagelos College of Physicians and Surgeons, Columbia University, New York, NY, USA; ^50^ La Follette School of Public Affairs, University of Wisconsin‐Madison, Madison, WI, USA; ^51^ Department of Neurology, Columbia University Medical Center, New York City, NY, USA; ^52^ Department of Statistics, University of Wisconsin‐Madison, Madison, WI, USA; ^53^ Department of Biostatistics and Medical Informatics, University of Wisconsin‐Madison, Madison, WI, USA; ^54^ Department of General Internal Medicine, University of Washington School of Medicine, Seattle, WA, USA; ^55^ Vanderbilt Memory and Alzheimer's Center, Vanderbilt University School of Medicine, Nashville, TN, USA

## Abstract

**Background:**

Despite evidence that Alzheimer's Disease (AD) is a highly heritable disease, there remains substantial “missing” heritability, likely due to the clinical and neuropathologic heterogeneity inherent in the disease. Here, we leverage sensitive longitudinal cognitive measures as endophenotypes in a rare variant analysis to identify novel genetic drivers of cognitive decline in aging and disease.

**Method:**

We leveraged 8 cohorts of cognitive aging with whole genome sequencing data from the AD Sequencing Project to conduct rare variant analyses of multiple domains of cognition (*N* = 8,481; mean age=73; 56% female; 52% cognitively unimpaired). Harmonized scores for memory, executive function, and language were derived using confirmatory factor analysis models. Longitudinal scores were generated for each domain using linear mixed model regressions. Participants of European ancestry inferred using SNPweights and 1000G reference panel were included. Variants included had a minor allele frequency < 0.01 and were annotated as a high or moderate impact SNP using VEP. We performed SKAT‐O testing for genes with at least two variants contributing and with a minimum aggregate minor allele count >10. All tests were adjusted for sex, baseline age at cognitive assessment, sequencing center and platform, and the first 5 principal components of genetic ancestry. Correction for multiple comparisons was completed using the false discovery rate (FDR) procedure.

**Result:**

We identified 9 genes associated with our cognitive domains. Two genes (*APOE, PSEN1*) were associated with baseline memory (both p_FDR_=0.07), one (*PEDS1‐UBE2V1*) with baseline language (p_FDR_=0.01), and six (*HPN, HPN‐AS1, GAB1, CXCL3, SIGIRR, PLA2G4A*) with executive function decline (p_FDR_ range=0.01‐0.08). *SIGIRR, PLA2G4A*, and *HPN* all had high impact variants contributing to the gene score that were significantly associated with executive function decline.

**Conclusion:**

These results highlight novel rare variants associated with cognition. *GAB1* is an AGORA nominated gene target for potential AD treatment. Decreased expression was found in cholinergic neurons in AD patients and decreased learning and memory in a mouse model of AD. *PLA2G4A* has increased expression in AD patients that is evident in early stages but is decreased in healthy aging brains. Future work will incorporate other ancestries.